# Vaspin identified as a DNA‐binding serpin with functional consequences for protease inhibition

**DOI:** 10.1111/febs.70270

**Published:** 2025-09-21

**Authors:** Kevin Möhlis, Abibe Useini, Heike Betat, Sonja Bonin, Helen Broghammer, Rima Nuwayhid, Stefan Langer, Mario Mörl, Norbert Sträter, John T. Heiker

**Affiliations:** ^1^ Helmholtz Institute for Metabolic Obesity and Vascular Research (HI‐MAG) of the Helmholtz Zentrum München at the University of Leipzig and University Hospital Leipzig Germany; ^2^ Institute of Bioanalytical Chemistry, Centre for Biotechnology and Biomedicine Leipzig University Germany; ^3^ Institute of Biochemistry, Faculty of Life Sciences Leipzig University Germany; ^4^ Division of Plastic, Aesthetic and Special Hand Surgery, Department of Orthopaedic, Trauma and Plastic Surgery University Hospital Leipzig Germany

**Keywords:** adipocytes, DNA, nucleus, polyphosphate, serpin

## Abstract

Vaspin is highly expressed not only in the skin but also in the liver and adipose tissue. It counteracts inflammation and oxidative stress in inflammatory skin diseases, obesity, and associated metabolic disorders, in part by inhibiting the kallikrein proteases KLK7 and KLK14. Vaspin binds the cell‐surface low‐density lipoprotein receptor‐related protein 1 (LRP1) with nanomolar affinity, and is rapidly internalized into adipocytes and other cells. We found intracellular vaspin partially localized in the nucleus. Since vaspin binds heparin and inorganic polyphosphates, we investigated the DNA binding of vaspin. Using DNA‐affinity chromatography and differential radial capillary action of ligand assays, we found high‐affinity binding to random sequences of single‐ and double‐stranded DNA for both vaspin and KLK7. Furthermore, KLK7 inhibition was accelerated fivefold in the presence of DNA molecules at least 40 bases in length. We previously identified the heparin‐binding site at a basic patch on the central beta‐sheet A of vaspin. In the current work, we determined the crystal structure of polyphosphate P45‐bound vaspin, which confirmed previously identified residues mutated to generate a nonheparin‐binding (NHB) vaspin variant. While NHB vaspin failed to bind heparin and polyP45, it still bound DNA with high affinity and accelerated protease inhibition. Mutation of closely spaced basic residues in helix A and helix G did not significantly alter DNA binding. In conclusion, we have identified vaspin as the second human DNA‐binding serpin. While the exact mode of the nonspecific interaction remains unclear, it accelerates protease inhibition and likely contributes to the nuclear localization observed for internalized vaspin and may allow for intracellular effects.

## Graphical abstract

Vaspin is a serpin that is expressed in skin, adipose tissue, and liver. It binds to single‐ and double‐stranded DNA with high affinity. This binding is unaffected by mutation of the known heparin‐binding site, accelerates the inhibition of the inflammatory protease KLK7, and may contribute to vaspin's nuclear localization. These findings establish vaspin as the second human DNA‐binding serpin and imply additional intracellular functions beyond the regulation of extracellular inflammation.

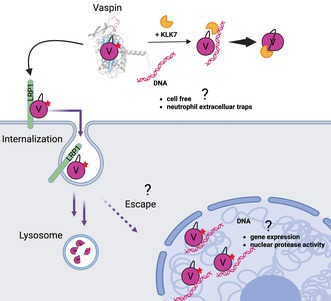

**Abbreviations**
ACT: antichymotrypsin
b: base(s)
cfDNA: cell‐free DNA
DRaCALA: differential radial capillary action of ligand assay
ds: double‐stranded
HCAEC: human coronary artery endothelial cells
kb: kilobase(s)
KLK7: kallikrein 7
LRP1: low‐density lipoprotein receptor‐related protein 1
MENT: myeloid and erythroid nuclear termination protein
NET: neutrophil extracellular traps
NHB: nonheparin‐binding
PIC: protease inhibitor complex
PIP: phosphatidylinositol phosphate
polyP: polyphosphate
RCL: reactive center loop
SAT: subcutaneous adipose tissue
SCCA‐1: squamous cell carcinoma antigen 1
SEC: serpin–enzyme complex
ss: single‐stranded
SVF: stromal vascular fraction
TAMRA: tetramethylrhodamine‐5(6)‐C2‐maleimide
WT: wild‐type

## Introduction

Vaspin (SERPINA12) is a serine protease inhibitor originally identified in the visceral adipose tissue of a rat model of type 2 diabetes [1]. It is highly expressed in skin, but also in other tissues and organs, including adipose tissue, liver, pancreas, placenta, and hypothalamus [1, 2, 3, 4, 5, 6]. Vaspin targets two proteases of the kallikrein family in KLK7 and KLK14 [6, 7]. In humans, circulating vaspin levels are associated with obesity and diabetes [8]. Vaspin exhibits anti‐inflammatory effects that contribute to improved glucose and lipid metabolism and overall metabolic health in mouse models of obesity [9, 10, 11].

We recently found that vaspin is actively internalized into adipocytes and other cells following the binding of the low‐density lipoprotein receptor‐related protein 1 (LRP1) [12]. While the receptor is recycled back to the cell membrane, a large part of vaspin was targeted for lysosomal degradation. However, we also observe that a fraction of vaspin seems to be localized in the nucleus. This has also been shown for other human serpins, such as the intracellular SERPINB3 (squamous cell carcinoma antigen 1; SCCA‐1) [13], but also for the secreted serpin antichymotrypsin (SERPINA3, ACT) [14]. While SERPINB3 does not bind DNA [15], DNA binding is speculated to underlie nuclear localization and accumulation of ACT [16]. ACT is the only human serpin shown to bind DNA and was originally isolated from serum using DNA‐affinity chromatography [16, 17]. Other serpins known to bind DNA include the bacterial chloropin and the avian nonhistone heterochromatin‐associated serpin, also called myeloid and erythroid nuclear termination protein (MENT) [18, 19]. Chloropin's interaction with DNA has been found to significantly enhance thrombin inhibition by up to 74‐fold. Similarly, MENT has been reported to promote protease inhibition in parallel to facilitating heterochromatin formation [20, 21]. Interestingly, DNA binding of ACT, which is sequence unspecific and exclusive for single‐stranded DNA, has no effect on ACT inhibitor activity against chymotrypsin [16]. Based on the known interactions of vaspin with cofactors, such as heparin, phospholipid species, and inorganic polyphosphate [22, 23], we speculated that vaspin may bind DNA.

To this end, we explored DNA‐binding properties of vaspin using DNA‐affinity chromatography and differential radial capillary action of ligand assay (DRaCALA) and further investigated the effects of DNA on target protease inhibition using single‐stranded DNA oligomers as well as double‐stranded plasmid DNA. DNA binding may explain subcellular localization of internalized vaspin and further enable intracellular biological activities yet to be explored.

## Results

### Internalized vaspin is in part transported into the nucleus

**Fig. 1:**
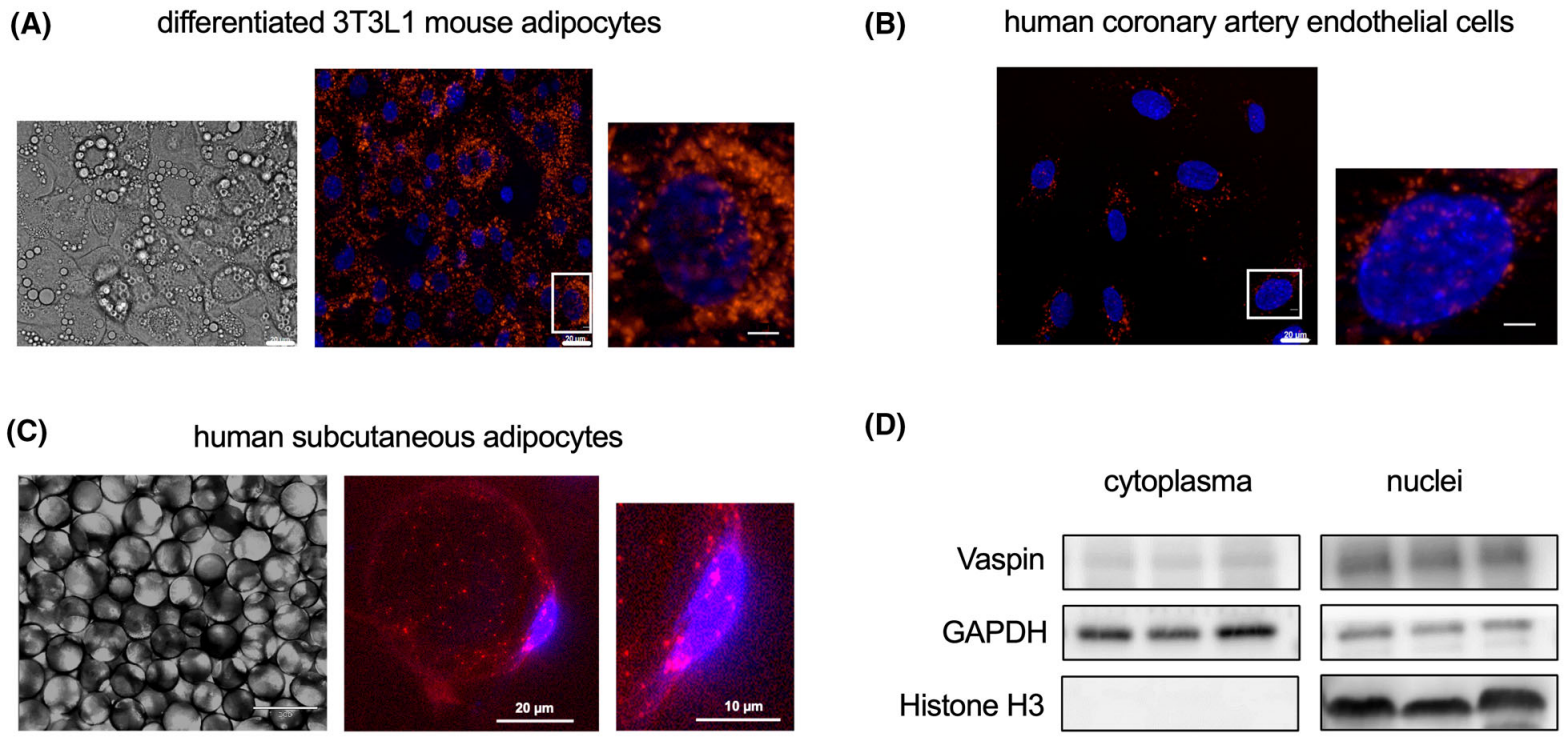
Nucleic uptake of vaspin in human adipocytes. (A, B) Fluorescence microscopy of differentiated 3T3‐L1 mouse adipocytes (A) and human coronary artery endothelial cells (B) after incubation with 100 nm TAMRA‐vaspin for 3 h (left—brightfield, middle (only in A) and right—fluorescence image and zoom of indicated area; scale bars: left, middle (only in A)—20 μm, right—5 μm). (C) Fluorescence microscopy of mature human adipocytes isolated from subcutaneous adipose tissue after incubation with 100 nm TAMRA‐vaspin for 30 min (left—brightfield, middle and right—fluorescence image of a single adipocyte and zoom of nucleus; scale bars: left—200 μm, middle—20 μm, right—10 μm). (D) Western blot analysis of cytoplasmic and nucleic fractions of differentiated adipocytes from the stromal vascular fraction (SVF) of human subcutaneous adipose tissue after incubated with 100 nm TAMRA‐vaspin for 30 min. Vaspin (top row) is present in cytoplasmic (mid row—enriched in GAPDH) and nucleic fractions (bottom row—containing histone H3). Blue signal: DAPI nuclear stain, red signal: TAMRA‐vaspin. Data are representative of three (A, B) or two (C, D) independent experiments.

We have previously reported vaspin internalization into cells mediated by the LRP1 [12]. Analysis of fluorescence microscopy images of differentiated 3T3‐L1 mouse adipocytes and human coronary artery endothelial cells (HCAEC) incubated with fluorescently labeled vaspin for 3 h revealed substantial nuclear fluorescence in addition to the endo‐ and lysosomal distribution of internalized vaspin (Fig. 1A,B). This suggests that a fraction of internalized vaspin may be targeted to the nucleus. To confirm these findings, we also incubated human mature adipocytes from subcutaneous adipose tissue with fluorescently labeled vaspin and again observed distinct nuclear fluorescence signals (Fig. 1C). To further validate the nuclear localization, we performed subcellular fractionation of differentiated human SVF‐derived adipocytes after incubation with fluorescently labeled vaspin for 30 min. We clearly detected vaspin in both cytoplasmic and nuclear fractions, with the majority appearing to be located in the nucleus (Fig. 1D). Taken together, we find that internalized vaspin is not only destined for lysosomal degradation, but a substantial fraction is transferred to the nucleus. As hypothesized for ACT, this may be enabled by DNA binding of vaspin.

### Vaspin and KLK7 bind DNA with nanomolar affinity

**Fig. 2:**
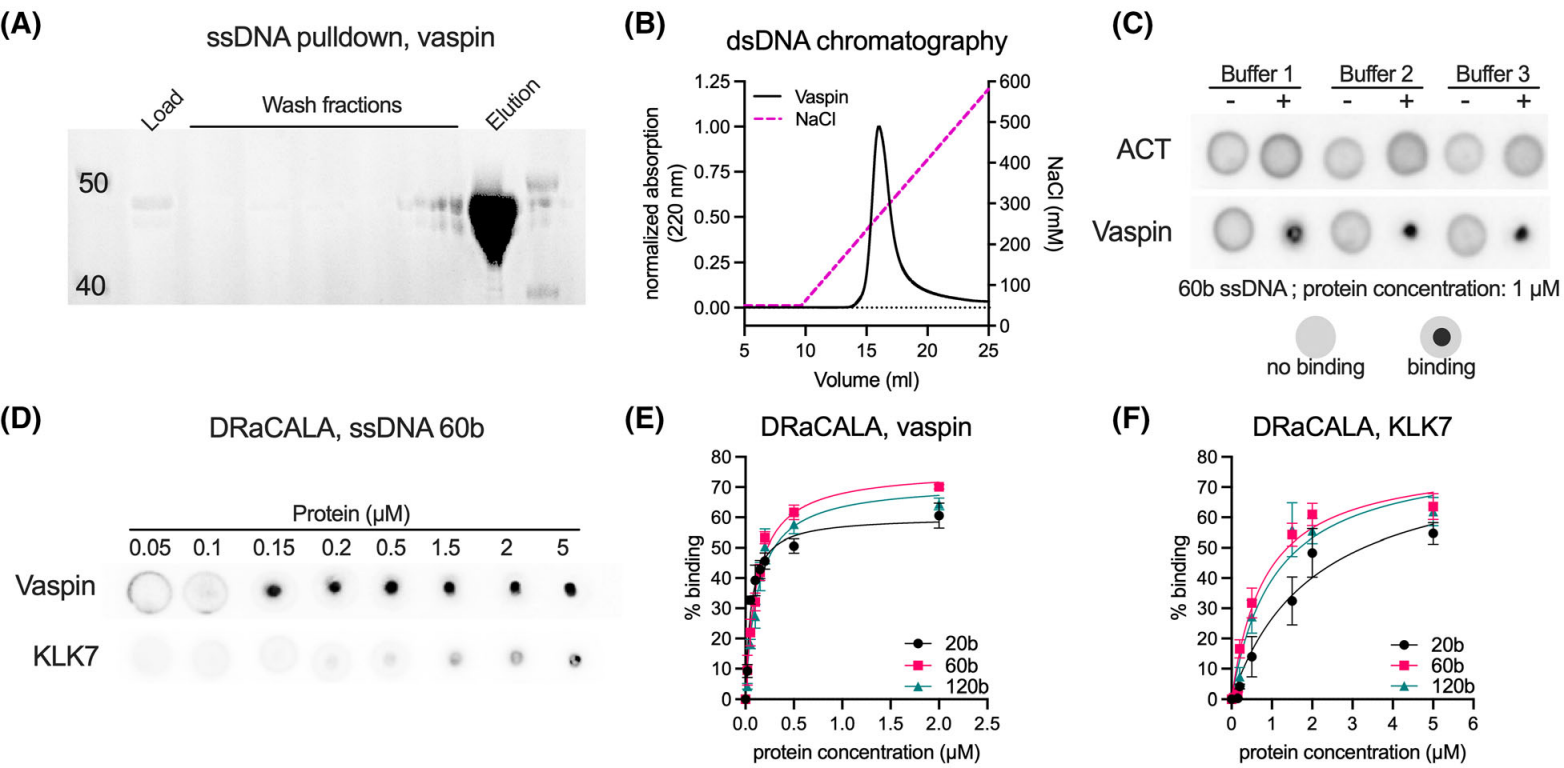
Vaspin binds DNA with nanomolar affinity. (A) Coomassie‐stained SDS/PAGE gel analyzing fractions of a pull‐down experiment using single strand (ss)DNA‐cellulose beads and recombinant human vaspin. (B) DNA‐affinity chromatography analysis of vaspin using double‐strand (ds)DNA‐cellulose with NaCl‐gradient elution. The same WT vaspin curve was used in Fig. 5A,H. (C) Buffer screening for DRaCALA‐based determination of human antichymotrypsin (ACT) and vaspin (1 μm for both) binding to radiolabeled 60 base ssDNA (50 nm). Representative DRaCALA images are shown and buffer compositions are stated in the Materials and methods section. (D) Representative DRaCALA images analyzing vaspin and kallikrein 7 (KLK7) binding to 60 base ssDNA in Buffer 2 (20 mm Tris/HCl, 15 mm NaCl, pH 7.8,). (E, F) *K*_D_ values were determined by nonlinear regression (one site—specific binding) plotting % binding versus protein concentration and are indicated by solid lines. Data are presented as mean + SEM of *n* = 3 technical replicates.

To investigate a possible interaction of vaspin with DNA, we first performed a pull‐down assay using ssDNA‐cellulose beads. Indeed, after a wash step with 150 mm NaCl, WT vaspin was eluted from the beads with Laemmli buffer, indicating a significant interaction (Fig. 2A). This was further confirmed by DNA‐affinity chromatography using dsDNA cellulose. WT vaspin eluted in a single peak at a concentration of 268 mm NaCl (Fig. 2B). To determine specific binding affinities, we used the DRaCALA approach [24] to test the interaction of WT vaspin and antichymotrypsin (ACT) with a radiolabeled 60 base ssDNA oligomer. This method is based on the separation of protein‐bound ligand, that is immobilized on the nitrocellulose membrane at the site of application, from free ligand, that is washed out with the solvent by capillary action (Fig. 2C). We first screened buffers, including low salt concentrations and a previously reported buffer (Buffers 1–3, see Materials and methods) [16]. Notably, we did not obtain binding signals from ACT toward 60 base ssDNA under all conditions tested. However, WT vaspin gave specific and strong signals in all buffers (Fig. 2C). Subsequent experiments were performed in Buffer 2 (20 mm Tris/HCl, pH 7.8, 15 mm NaCl), and we determined the binding affinities of vaspin and the target protease KLK7 using ssDNA oligomers of 20, 60, and 120 bases in length (Fig. 2D, for 60 b ssDNA). WT vaspin exhibited nanomolar binding affinities with *K*_D_ ranging from 59 to 128 nm, decreasing with oligomer length, whereas KLK7 showed an overall weaker binding with *K*_D_ ranging from 815 to 2066 nm (Fig. 2E,F, Table 1). These results demonstrate high affinity DNA binding for both vaspin and its target protease KLK7.

**Table 1:** Binding affinities (*K*_D_) of wild‐type (WT) vaspin, NHB vaspin and KLK7 for 20, 60, or 120 base ssDNA oligomers. Data are presented as best‐fit and 95% confidence interval from at least *n* = 3 independent experiments.

	*K*_D_ (nm)		
	20 base	60 base	120 base
WT vaspin	59 (41–82)	117 (91–149)	128 (94–172)
KLK7	2066 (1049–4439)	815 (553–1216)	1066 (635–1841)
NHB vaspin	269 (188–384)	188 (115–297)	178 (93–325)

### DNA accelerates the inhibition of KLK7 by vaspin

**Fig. 3:**
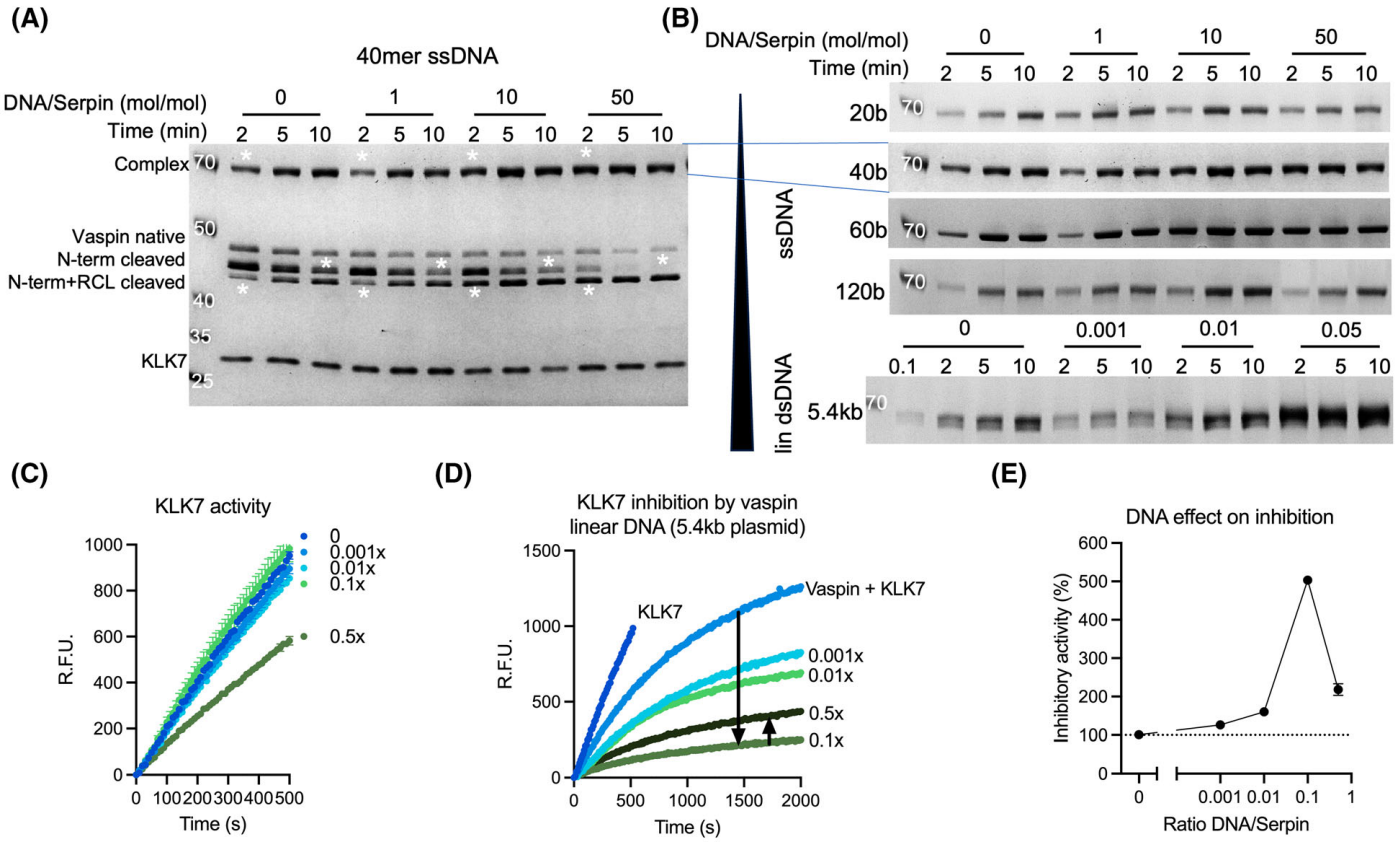
DNA binding accelerates KLK7 inhibition by vaspin. (A) Analysis of DNA‐accelerated complex formation of vaspin and KLK7 in the presence of increasing concentrations of 40 base ssDNA, including a control without DNA, after 2‐, 5‐, and 10‐min reaction times. Coomassie Blue‐stained SDS gel of vaspin and KLK7 incubated at a fixed molar ratio (1 : 1) and with increasing concentrations of DNA (1–50‐fold vaspin). Increasing band intensities of complexed and N‐terminally cleaved (after 2 min) and decreasing band intensity of RCL‐cleaved vaspin (after 10 min) are indicated by asterisks and confirm the accelerated interaction of serpin and protease in the presence of DNA. From top to bottom the labels describe: Vaspin‐KLK7 complex, native vaspin, N‐terminally cleaved vaspin, N + C‐terminally cleaved vaspin, and KLK7. (B) Analysis of DNA‐accelerated complex formation of vaspin and KLK7 in the presence of increasing concentrations of 20, 40 (as in A), 60, and 120 base ssDNA as well as 5.4‐kb linear dsDNA and a control without DNA after 2‐, 5‐ and 10‐min reaction times. Only the vaspin‐KLK7 complex band is shown. (C) Analysis of KLK7 proteolytic activity in the presence of increasing concentrations of 5.4‐kb linear dsDNA using a fluorogenic peptide substrate. (D) Continuous monitoring of the vaspin‐KLK7 inhibition reaction in the presence of increasing concentrations of linear dsDNA measuring residual activity using a fluorogenic substrate peptide. Arrowheads indicate concentration‐dependent reduction of KLK7 activity in the presence of DNA. (E) DNA‐induced acceleration of vaspin inhibition of KLK7 in the presence of DNA, corrected for KLK7 activity in the presence of corresponding DNA concentration. The bell‐shaped curve suggests the bridging or template effect as the major contribution to DNA‐accelerated vaspin reactivity. Gels are representative of at least *n* = 2 independent experiments, data in (D), (E) are represented as mean + SEM of *n* = 5 technical replicates. R.F.U., relative fluorescence units.

The binding of both vaspin and KLK7 to DNA would enable DNA to serve as a bridging molecule, accelerating the inhibition reaction of serpin and protease. To determine the effect of ssDNA and dsDNA on the inhibition of KLK7 by vaspin, we analyzed serpin–enzyme complex (SEC) formation in the presence and absence of DNA by SDS/PAGE (Fig. 3A,B). Vaspin and KLK7 at a molar ratio of 1 : 1 were incubated with increasing concentrations of ssDNA of different lengths as well as with linearized dsDNA (5.4 kb) for the indicated time. The SEC (1) and cleaved vaspin (4) band intensities increase over time and with increasing DNA concentration, while native vaspin (2) band intensity decreases. White asterisks indicate the bands of decreasing partially cleaved vaspin (3) and increasing SEC (1) formation and fully cleaved vaspin (4) generation, together demonstrating the accelerated interaction between vaspin and KLK7.

The bridging effect with accelerated complex formation was most evident for the linearized dsDNA (Fig. 3B). Incubation with the 20‐base oligomer did not affect SEC formation, suggesting a minimum requirement of 40 bases to bridge serpin and protease. We then used a continuous method [25] to monitor the effect of linearized dsDNA on the inhibition reaction over time. We first examined KLK7 activity in the presence of increasing concentrations of dsDNA (Fig. 3C). Notably, the highest DNA concentration (0.5× or 200 nm) resulted in a decrease of ~ 40% in the overall activity of KLK7 (Fig. 3C). To calculate the overall inhibition, we used the endpoint fluorescence values (45 min), adjusted for the effect of DNA on KLK7 activity (at 45 min), and normalized these adjusted fluorescence values to the Vaspin‐KLK7 reaction without DNA. Together with vaspin (molar ratio 1 : 1) and increasing concentrations of dsDNA, the inhibition reaction was significantly accelerated, resulting in an up to a fivefold increase in the presence of 0.1‐fold linearized dsDNA (Fig. 3D,E). Taking into account the reduced activity of KLK7, a further fivefold increase in dsDNA concentration abolished the reaction‐accelerating effect, resulting in an overall bell‐shaped concentration‐response curve.

### The crystal structure of polyP‐bound vaspin confirms binding site on central β‐sheet A

**Fig. 4:**
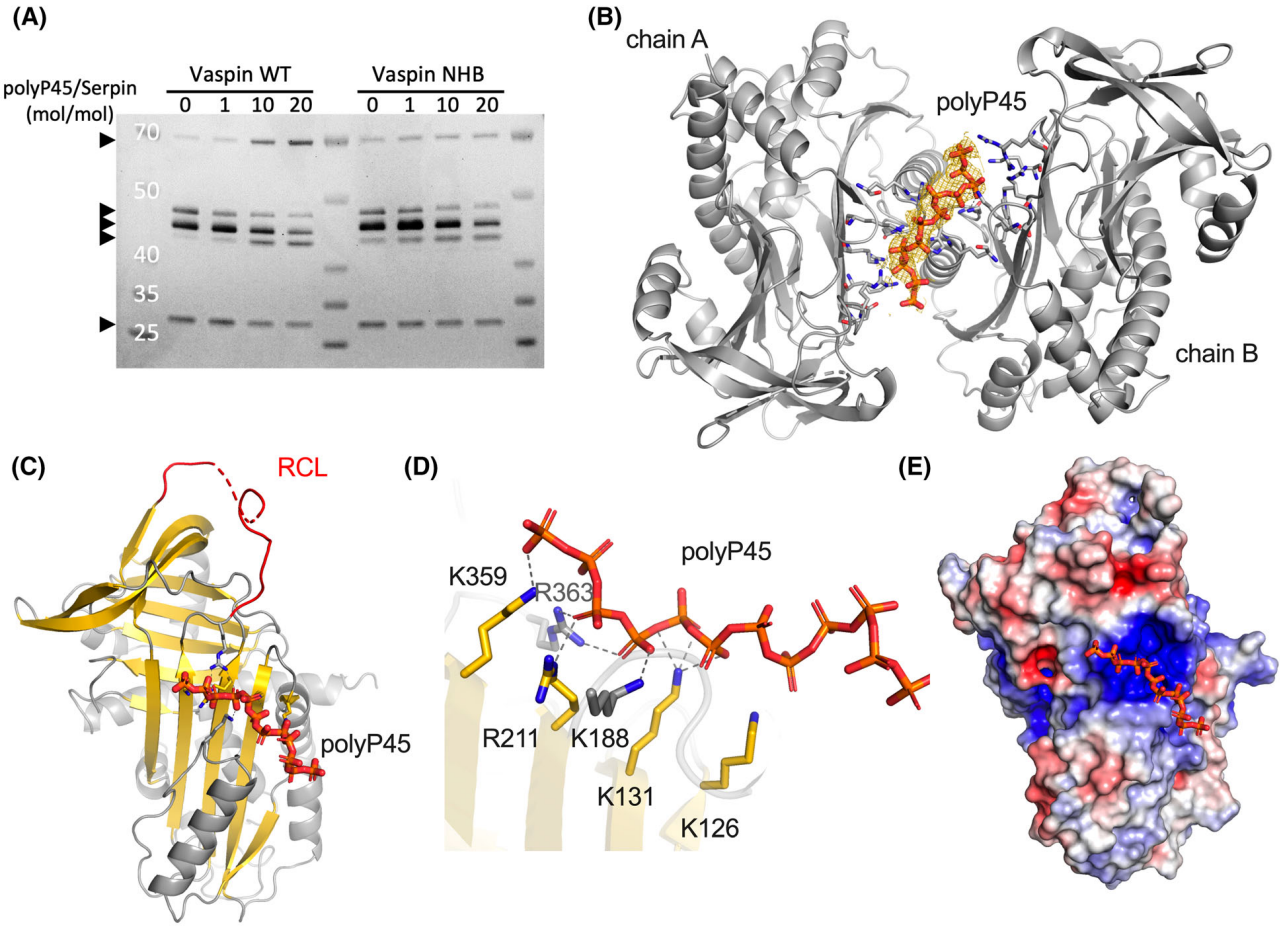
Crystal structure of polyphosphate polyP45‐bound vaspin. (A) Analysis of DNA‐accelerated complex formation of vaspin [wild‐type (WT) and nonheparin‐binding (NHB)] and KLK7 (1 : 1 molar ratio) in the presence of increasing concentrations of polyP45 (0 to 20‐fold vaspin) and a control without DNA after 2‐min reaction time by SDS/PAGE. WT vaspin shows accelerated complex formation and this is lost for the NHB variant. Arrowheads indicate bands from top to bottom corresponding to (1) Vaspin‐KLK7 complex, (2) native vaspin, (3) N‐terminally cleaved vaspin, (4) N + C‐terminally cleaved vaspin, (5) KLK7. (B) Crystal structure of polyP45‐bound WT vaspin (pdb id: 9IH0). Twelve phosphate residues were modeled into the 2*F*_o_ − *F*_c_ density (yellow). The polyphosphate binds between the two protein chains in the asymmetric unit. The view is along the noncrystallographic twofold symmetry axis. (C) Overview of the binding site of polyP45 to vaspin. The reactive center loop is shown in red. (D) Close‐up view of the residues interacting with the polyphosphate chain. (E) Electrostatic potential mapped to the molecular surface [color coded from −5 kT/e (red) to +5 kT/e (blue)]. The polyphosphate chain is located at the basic patch at the central beta‐sheet A of vaspin. Figures B–E were generated using pymol.

Previous studies have shown that larger inorganic polyphosphates bind vaspin and accelerate the complexation rate with KLK7 [23], similar to our findings with WT vaspin and DNA. Mutations of lysine and arginine residues in beta‐sheet A of vaspin identified by a selective labeling approach [22] resulted in a nonheparin‐binding variant (NHB vaspin; K131A/K188A/R211A/K359A/K363A; [23]) that also abolished polyphosphate binding (Fig. 4A). To further characterize and locate potential shared key residues in vaspin binding to inorganic polyphosphate, vaspin was co‐crystalized with polyP3 or polyP45, and crystal structures were determined. Crystal structures of vaspin were previously characterized in the uncleaved [6] and cleaved [26] states. The vaspin‐P3 and vaspin‐P45 structures were resolved at resolutions of 2.12 and 2.26 Å, respectively. While no ligand‐bound crystals were obtained for vaspin with polyP3, a co‐crystal structure was obtained for vaspin with polyP45. Vaspin with polyP45 crystallized in a C2 space group, which contains a similar arrangement of two vaspin molecules (chains A and B) in the asymmetric unit as the previously determined structure of uncleaved vaspin (pdb id: 4IF8) [6]. Both vaspin molecules are uncleaved and superimpose closely with the unliganded vaspin structure 4IF8 (root mean square deviation of 0.684 Å for chains A of the asymmetric unit). Polyphosphate polyP45 binds between chain A and B (Fig. 4B). The electron density allowed for modeling 12 phosphates of the polyP45 chain. The ligand in this position was refined with 0.52 occupancy and an average B‐factor of 72.85 Å^2^. The relatively high B‐factor value is comparable to the surrounding residues. Six interacting residues were identified (Fig. 4C,D; K126, K131, K188, R211, K359, and R363). These residues include all the basic residues mutated in the NHB vaspin variant plus K126, located directly in the basic patch of the protein (Fig. 4E). The electron density indicates that polyphosphate polyP45 also adopts a second (alternative) conformation. However, the density is not defined well enough to model this conformation.

### Mutation of the heparin‐ and polyP‐binding site does not affect DNA binding

**Fig. 5:**
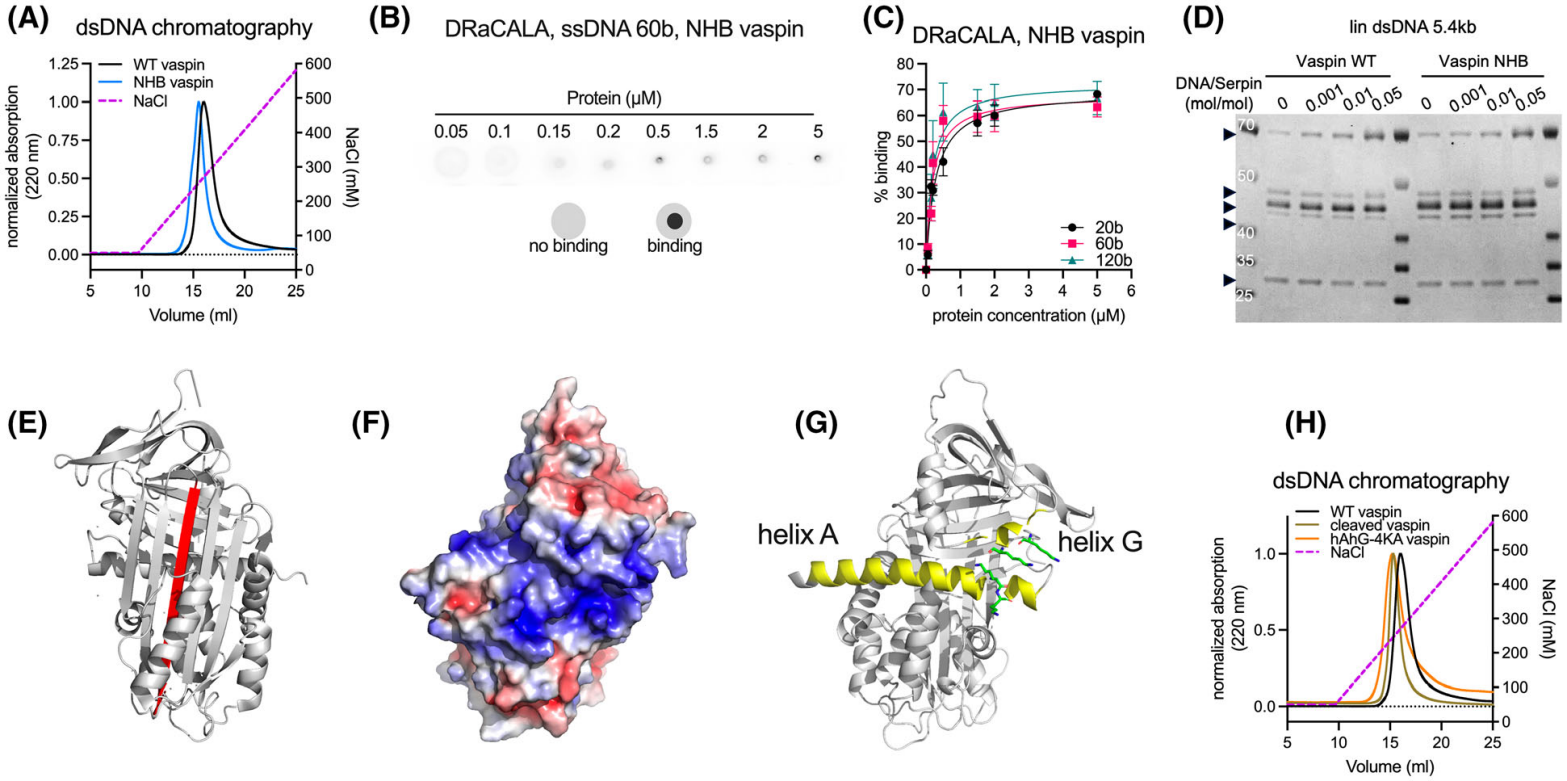
Nonheparin‐binding (NHB) vaspin mutant still binds DNA. (A) DNA‐affinity chromatography analysis of wild‐type (WT) and NHB vaspin using dsDNA‐cellulose and NaCl‐gradient elution. (B) DRaCALA‐based determination of NHB vaspin (1 μm) binding to radiolabeled 60 base ssDNA (50 nm) in Buffer 2 (20 mm Tris/HCl, pH 7.8, 15 mm NaCl). (C) *K*_D_ was determined by nonlinear regression (one site—specific binding) plotting % binding versus protein concentration and is indicated by solid lines. Data in (B, C) are presented as mean + SEM of *n* = 3 technical replicates. (D) Analysis of DNA‐accelerated complex formation of WT or NHB vaspin and KLK7 in the presence of increasing concentrations of 5.4‐kb linear dsDNA and a control without DNA after 2‐min reaction time by SDS/PAGE. Arrowheads indicate bands from top to bottom corresponding to: (1) Vaspin‐KLK7 complex, (2) native vaspin, (3) N‐terminally cleaved vaspin, (4) N + C‐terminally cleaved vaspin, (5) KLK7. (E) Crystal structure of cleaved vaspin (pdb id: 5EI0) with the inserted reactive center loop (RCL) highlighted in red. (F) Electrostatic potential at the molecular surface [color coded from −5 kT/e (red) to +5 kT/e (blue)] on the backside of WT vaspin (pdb id: 4IF8) reveals a basic patch. (G) Crystal structure of native vaspin (pdb id: 4IF8) with helix A and G highlighted in yellow and lysine residues (K61/K62/K281/K285; mutated in the hAhG‐4KA vaspin variant) shown as sticks. Figures E–G were generated using pymol. (H) DNA‐affinity chromatography of WT, cleaved, and hAhG‐4KA vaspin using dsDNA cellulose and NaCl‐gradient elution. Data (A, D, and H) are representative of two independent experiments.

The polyP45‐bound crystal structure of vaspin ultimately confirmed the binding of polyP to this basic patch of lysine and arginine residues on top of the central β‐sheet A (Fig. 4C), and consistently the NHB vaspin variant did not show polyP‐accelerated complex formation with KLK7 (Fig. 4A). We hypothesized that DNA binding would occur at the same binding site. However, when we repeated the DNA‐binding experiments with the NHB vaspin variant, we did not observe any effect of the mutated residues. In contrast to polyphosphate binding, DNA binding of NHB vaspin was comparable to that of WT vaspin in DNA‐affinity chromatography (Fig. 5A; NHB: 253 mm NaCl, WT: 268 mm NaCl). Similarly, DRaCALA with radiolabeled 20‐, 60‐, and 120‐base ssDNA oligomers showed nanomolar affinities (between 100 and 250 nm, Table 1) for all oligomers, with a fivefold lower affinity for the 20‐base oligomer, but less than a twofold reduction for the 60‐ and 120‐base oligomers (Fig. 5B,C, Table 1). Finally, complex formation with KLK7 in the presence of linear dsDNA also showed similar reaction acceleration for WT and NHB vaspin (Fig. 5D).

To examine whether the reactive center loop (RCL) is involved in DNA binding, we tested cleaved vaspin in dsDNA chromatography (Fig. 5E,H). Additionally, we mutated four lysines (K61A/K62A/K281A/K285A, hAhG‐4KA vaspin variant), which are located in helices A and G on the backside of vaspin and form a basic stretch (Fig. 5F,G). However, the elution profiles for WT, cleaved vaspin, and hAhG‐4KA vaspin were similar (Fig. 5H: WT: 268 mm NaCl, cleaved: 246 mm NaCl, hAhG‐4KA: 245 mm NaCl), indicating that DNA binding is not mediated by the exposed RCL nor the basic stretch at helix A and G.

Kallistatin (SERPINA4) binds to heparin at or near helix H, with contributions from numerous basic residues [27]. These residues include R306, R307, R308, and R309 of the 310 helix H′, which is a unique insertion present only in the serpin kallistatin, two adjacent lysine residues (K312 and K313) close to the RCL, as well as R300 of helix H and R259 of s6B. Superposition of vaspin and kallistatin structures reveals that, except for residues of the unique insertion, R301 and R302 (close to the RCL), R294 and K296 (of helix H), and K254 (of s6B) are also present in vaspin. We have previously shown that these are not involved in the heparin binding of vaspin [22]. To assess whether these residues may be involved in DNA binding of vaspin, we generated two variants (K254A/R294A/K296A and K254A/R294A/K296A/R301A/R302A). Unfortunately, both were predominantly expressed as inclusion bodies, and purification of the soluble fraction via IMAC resulted in low amounts of only partially purified protein eluting at the 150 mm imidazole wash step (data not shown). Ultimately, these protein preparations were not suited for follow‐up DNA‐affinity chromatography or DraCALA assays.

These results demonstrate that vaspin interacts with polyphosphate polyP45 and DNA via distinct binding sites, potentially with contributions from multiple basic patches with equal affinity.

## Discussion

We have previously reported that the human serpin vaspin can bind to several membrane phospholipid species and inorganic polyphosphates [23]. Similar to heparin [22], sufficiently long linear orthophosphate polymers, such as polyP45, serve as a bridging molecule to accelerate vaspin inhibition of target proteases, such as KLK7 [23].

This study follows up on these findings and shows that vaspin and KLK7 can bind both single‐stranded and double‐stranded DNA with high affinity. Binding of DNA with a minimum length of 40 bases was required for both proteins to bind and resulted in significantly accelerated protease inhibition. Furthermore, DNA binding may also contribute to stabilization of the complex against dissociation.

DNA binding has so far only been reported for a single human serpin, alpha‐1‐antichymotrypsin (SERPINA3/ACT) [16, 17], but with striking differences when comparing the affinity, selectivity, and consequences of DNA binding to vaspin. Studies with recombinant rat ACT showed that DNA binding was exclusive to double‐stranded DNA and did not show sequence specificity. Furthermore, there was no effect of DNA binding on the inhibition of the target protease (chymotrypsin) [16], suggesting no conformational changes that accelerate the inhibition reaction.

Given the random sequence of shorter DNA oligomers and of the linearized plasmid DNA used in our study, we conclude that DNA binding of vaspin represents no sequence‐specific DNA‐protein interactions based on ionic interaction of basic residues with the phosphate backbone of the DNA. Based on our previous data and the crystal structure of polyP‐bound vaspin, we speculate that this would occur at the same site and residues that we found to be critical for both heparin and polyP binding. However, our mutant vaspin, which lacks five of the six basic residues, showed remarkably similar results in all aspects of DNA binding, including nanomolar affinity for ssDNA oligomers and accelerated complex formation with KLK7 in the presence of dsDNA. This suggests a molecular interaction different from that of heparin and polyPs.

For ACT, significant contributions of three consecutive lysine residues (210–212) and the C terminus (K391/K396) have been described. While vaspin lacks this triple lysine motif, future studies could focus on C‐terminal mutagenesis of the corresponding K406/K414 residues.

For the avian chromatin‐associated serpin MENT, DNA binding induces conformational changes that enhance protease inhibition activity [20]. For MENT, DNA binding is primarily mediated by an interhelical extension called M‐loop, with contributions from residues of the D‐ and E‐helices [21, 28]. This feature is unique to the MENT serpin.

To further investigate potential DNA‐binding residues, we tested cleaved vaspin to determine whether the insertion of the RCL loop into β‐sheet A affects DNA binding. We also screened for other larger basic patches and identified one on the backside of helix A, where we mutated four lysine residues to alanine (hAhG‐4KA vaspin). Interestingly, neither the cleaved form nor the mutated basic patch on helix A and G altered the elution salt concentration during dsDNA chromatography (WT: 268 mm NaCl, cleaved: 246 mm NaCl, 4K: 245 mm NaCl). The exact location of DNA binding to vaspin thus remains unclear but may also comprise both basic patches together exhibiting similar affinities. Contributions of basic residues resembling the kallistatin heparin‐binding site could not be further investigated due to misfolding. However, with the positioning of this heparin‐binding site in kallistatin, just eight saccharide units were sufficient to bridge the serpin and thrombin and accelerate complex formation. In our experiments, only DNA oligos of more than 40 bases in length were able to bridge and accelerate complex formation. Therefore, the binding sites of vaspin and KLK7 likely are farther apart, indicating that the kallistatin‐like binding site is not primarily involved in DNA binding.

The cellular consequences or biological relevance of human serpin–DNA interactions remain unknown. Although ACT is a secreted serpin, it has been found in the nuclear fraction of neuronal and cancer cells [29, 30]. Since there was no evidence of sequence specificity for ACT DNA binding, it was speculated that nonspecific DNA binding may simply allow for increased nuclear levels of the serpin and/or protect from nontarget proteases by blocking sensitive/protease‐susceptible sites [31]. With this in mind, we analyzed the subcellular localization of fluorescently labeled vaspin after internalization, focusing on the nucleus. Indeed, we found distinct nuclear fluorescence signals in mouse and human adipocytes and endothelial cells. Subcellular fractionation of differentiated human adipocytes incubated with vaspin even suggested that not only a fraction of vaspin enters the nucleus. The exact mechanism underlying the nuclear translocation of vaspin remains unknown. Some serpins, such as maspin (SERPINB5), carry nuclear localization signal (NLS) sequences and are thought to translocate to the nucleus following a conformational change that exposes the NLS [32]. Analysis of the vaspin sequence using cNLS mapper [33] did not indicate the presence of a NLS (data not shown). Other potential nuclear import mechanisms include passive diffusion of small proteins (< 40 kDa) or binding to nuclear transport proteins [34]. Whether the nuclear presence of vaspin somehow leads to altered gene transcription or regulates nuclear proteases remains to be investigated.

However, there are circumstances that a secreted and circulating serpin may come into contact with extracellular DNA. Recent studies have investigated the release of cell‐free DNA (cfDNA) in obesity and have shown that elevated plasma levels of ss/dsDNA are observed in both mice and humans. Furthermore, visceral adipose tissue was shown to be a major source of cfDNA, and cfDNA levels correlated with visceral obesity and insulin resistance [35]. In addition, neutrophils and other types of immune cells can actively release DNA to form neutrophil extracellular traps (NETs) in an event called NETosis. The composition of NETs consists primarily of double‐stranded DNA (dsDNA) and anti‐inflammatory proteins (peptides, proteases, and inhibitors, guanosine histones) as a critical component of antimicrobial defense [36]. Excessive NETosis has been suggested to contribute to or even drive infectious, inflammatory, and autoimmune diseases and has also been associated with increased leukocyte and neutrophil numbers in obesity [37]. It is tempting to speculate that increasing levels of cfDNA may enhance vaspin inhibitory activity, driving the anti‐inflammatory action of recombinant or transgenic vaspin previously shown in adipose tissue and liver of diet‐induced obese mice [11, 38].

In conclusion, our study reveals that vaspin is a second human DNA‐binding serpin that binds nonspecifically to single‐ and double‐stranded DNA with high affinity and accelerates its proteolytic activity toward KLK7. In addition, the nuclear localization of internalized vaspin extends its range of action and may lead to intracellular effects. However, while the crystal structure of vaspin‐bound polyP45 confirmed the binding mode previously identified for heparin, mutation of the binding site did not affect DNA binding, suggesting a different binding mode that requires further investigation.

## Materials and methods

### Materials and recombinant proteins

Expression, purification, cleavage, and fluorescent labeling (where indicated) of recombinant human vaspin and its variants, as well as the functional assay with recombinantly expressed human KLK7, were carried out as previously described [12, 22].

DNA molecules used in this study were the linearized double‐strand pcDNA3.1+/C‐DYK plasmid and single‐strand (ss) DNA sequences derived from this plasmid:

20 bases (GACGGATCGGGAGATCTCCC);

40 bases (GACGGATCGGGAGATCTCCCGATCCCCTATGGTGCACTCT);

60 bases (GACGGATCGGGAGATCTCCCGATCCCCTATGGTGCACTCTCAGTACAATCTGCTCTGATG);

120 bases (GACGGATCGGGAGATCTCCCGATCCCCTATGGTGCACTCTCAGTACAATCTGCTCTGATGCCGCATAGTTAAGCCAGTATCTGCTCCCTGCTTGTGTGTTGGAGGTCGCTGAGTAGTGCG).

Linearization was performed using the restriction enzyme BamHI (#ER0055; Thermo Fisher Scientific, Waltham, MA, USA). BamHI (10 U) was incubated with 5 μg plasmid DNA for 30 min at 37 °C. Subsequently, the enzyme was inactivated at 80 °C for 5 min.

### DNA pull‐down assay

ssDNA‐cellulose beads (#IBODNACSO1S1GM; Innovative Research, Novi, MI, USA) were hydrated and washed with buffer (150 mm NaCl and 10 mm Tris) before the pull‐down assay. One hundred microliters of swollen beads and two hundred micrograms of recombinant vaspin were incubated overnight at 4 °C while rotating. The beads were centrifuged, washed five times with 500‐μL wash buffer, and bound protein was eluted using reducing SDS sample buffer. Samples were taken from the supernatant for each step and analyzed by SDS/PAGE.

### DNA‐affinity chromatography

dsDNA‐Cellulose beads (#IBODNACSO2S1GM; Innovative Research) were hydrated in water for a few minutes and loaded onto a 1‐mL chromatography column. The column was washed with equilibration buffer (10 mm Tris, 10 mm NaCl, 1 mm EDTA, 50 mm NaCl, pH 7.8), loaded with 250 μg recombinant protein, and incubated for 30 min at 4 °C. After repeated washing with equilibration buffer, bound protein was eluted by gradient elution using NaCl.

### Differential radial capillary action of ligand assay (DRaCALA)

The DRaCALA assay [24] was performed to test the binding of vaspin, ACT, and KLK7 to single‐stranded DNA oligos of different lengths (20, 60, and 120 bases). This method is based on the separation of free ligand from protein–bound ligand on a nitrocellulose membrane. To evaluate the impact of different buffer conditions, 50 nm of a ^32^P‐5′‐end labeled 60 base ssDNA oligonucleotide was incubated with 1 μm WT vaspin or 1 μm ACT, respectively, in one of the following buffers: Buffer 1 (20 mm Tris/HCl pH 7.8, 150 mm NaCl); Buffer 2 (low salt: 20 mm Tris/HCl pH 7.8, 15 mm NaCl); and Buffer 3 (20 mm Tris/HCl pH 7.8, 15 mm KCl, from Naidoo *et al*. [16]). For *K*_D_ determination, vaspin (WT and NHB) or recombinant human KLK7 were incubated in Buffer 2 with 50 nm labeled ssDNA oligomers (20, 60, and 120 bases) in Buffer 2 at protein concentrations ranging from 0 to 2 μm for vaspin (WT and NHB) or 0 to 5 μm for KLK7, respectively. All reactions were performed in a final volume of 10 μL for 10 min at 20 °C. For the DRaCALA, 2 μL of each sample solution were spotted onto a dry nitrocellulose membrane (Cytiva, Freiburg, Germany). The membrane rapidly immobilized proteins along with bound DNA at the spotting site, while unbound DNA diffused away. Autoradiography was used to distinguish between bound and unbound DNA. Values for bound DNA (% binding) from at least three independent experiments were plotted against the protein concentration. *K*_D_ values were determined using nonlinear regression analysis (one site—specific binding) using graphpad prism 10 (GraphPad Software, Boston, MA, USA).

### Kinetic analysis

To determine the rate of inhibition of the vaspin‐KLK7 interaction, a continuous method with a 10‐fold molar excess of vaspin was used as previously described [25]. Briefly, 200 μg·mL^−1^ KLK7 (#757004; BioLegend, San Diego, CA, USA) was activated using 20 μg·mL^−1^ bacterial thermolysin (#3097‐ZN; R&D, Minneapolis, MN, USA) and incubated for 2 h at 37 °C. The reaction was stopped by the addition of EDTA to a final concentration of 100 mm. For a continuous monitoring of the KLK7 inhibition reaction, 30 μm of KLK7‐substrate, NFF3 (Mca‐RPKPVE(Nva)WR(K(DNP))‐NH_2_), was added to a reaction mixture of 50 ng activated KLK7, a 10‐fold molar excess of vaspin, and increasing concentrations of ssDNA oligomers (20, 40, and 120 bases) or linearized dsDNA plasmid. The fluorescence signal was monitored at excitation/emission wavelengths of 320/405 nm in kinetic mode over 45 min (measurement every 10 s). Fluorescence signals were plotted vs. reaction time for each tested condition. To estimate the acceleration of the inhibition reaction, we used endpoint fluorescence data (45 min) to determine the effect of DNA concentrations tested on KLK7 activity alone. Subsequently, endpoint measurements of vaspin‐KLK7‐DNA reactions were first corrected for effects on KLK7 activity and then used to calculate inhibitory activity (%) using the vaspin‐KLK7 reaction as reference (100% inhibitory activity).

### Complex formation analysis by SDS/PAGE

To investigate complex formation, recombinantly expressed human KLK7 and vaspin, or mutants thereof, were incubated at a protease/serpin ratio of 1 : 1 in TBS. At predetermined time points, samples containing 0.5 μg of vaspin were collected, and the reaction was stopped by immediate addition of reducing SDS sample buffer and 5 min of heating at 95 °C. Protein separation by SDS/PAGE was performed using 4–12% Bis‐Tris Plus precast gels and NuPAGE MOPS/SDS running buffer (Life Technologies, brand of Thermo Fisher Scientific). The gels were then stained with Coomassie Blue, and digitization and densitometric analysis were performed using the iBrightTM CL1500 Imaging System and genetools analysis software (Syngene, Cambridge, UK). To analyze the effect of polyP45 and DNA on the vaspin–KLK7 reaction, complex formation assays were performed as described above with various concentrations of polyP45, ssDNA (20, 40, 60, and 120 bases) or linearized dsDNA (pcDNA3.1+/C‐DYK), and the reactions were stopped immediately or after 2, 5, and 10 min of incubation.

### Protein crystallization and structure determination

Purified protein was concentrated to 10 mg·mL^−1^ in 20 mm Tris/HCl pH 7.8, containing 150 mm NaCl. For co‐crystallization of vaspin with polyphosphates, either polyP3 or polyP45 was added to the protein sample to a final concentration of 10 mm. Stock solutions of polyP3 and polyP45 were prepared in advance using the same protein buffer.

Crystallization trials for all samples were conducted based on previously established conditions [6]. Initial experiments yielded suitable crystals for vaspin–polyP3, whereas further optimization was required for co‐crystallization of the vaspin–polyP45 complex. To enhance crystal quality, an additive screen from Hampton Research was employed. These setups were performed in a 96‐well format plate using the sitting‐drop vapor diffusion method. Crystallization drops were prepared by mixing equal volumes of protein and reservoir solution (200 nL final volume), equilibrated against 100 μL reservoir. Drops were pipetted using a Mosquito Xtal3 pipetting robot (SPT Labtech, Melbourn, UK).

Conditions that yielded optimal crystals were manually reproduced in hanging drops at microliter scale. Drops comprising of protein and reservoir solution (1 μL each) were equilibrated against 500 μL reservoir solution. Vaspin crystals formed in a solution containing 10 mm polyP3, 9% (w/v) PEG 4000, 0.1 m ammonium sulfate, 0.1 m sodium citrate, pH 5.1. Crystals of vaspin–PolyP45 were obtained in the same buffer composition (without polyP3), supplemented with 5% (v/v) DMSO and 0.5% (w/v) n‐octyl‐ß‐d‐glucoside. Crystals reached a size of 80–150 μm within a week.

Single crystals were harvested and flash frozen in liquid nitrogen using cryo‐buffer (15% glycerol (v/v) in the respective crystallization condition). Crystallographic data were collected at EMBL beamline P14 at PETRA III of the DESY synchrotron (Hamburg, Germany).

Diffraction data processing, including indexing, integration, and scaling, was carried out with xds (version 30 June 2023) [39] and staraniso (version 2.3.74) [40], as implemented in ispyb [41]. The structures were solved by molecular replacement using phaser (version 2.8.3) [42] and ccp4i2 (version 8.0.015). As the search model for this step, the structure with pdb id: 4IF8 [6] was used. Initial refinement, followed by jelly‐body refinement, was conducted with refmac version 5.8.0425 [43]. Models were completed in coot (version 0.9.8.93), and final refinement steps were done in phenix (version 1.21.2) [44]. The ligand restraints were generated using grade webserver (version 1.7.0rci, accessed on 16th Sept 2024). Data collection and refinement statistics are listed in Table 2. Figures were generated with pymol (version 3.0.2), and the electrostatic potential was generated by program apbs (version 1.5) [45].

**Table 2:** Diffraction data and refinement statistics.

Compound	Vaspin × polyP45
PDB entry ID	9IH0
Data collection	
Source	Beamline P14
Wavelength (Å)	0.9762
Resolution (Å)	101.08–2.26 (2.48–2.26)
Resolution aniso (Å)	2.840, 2.259, 2.378
Space group	C 1 2 1
Unit cell dimensions (Å; °)	143.26, 147.98, 62.46; 90, 104.949, 90
Unique reflections	40 630 (2032)
Multiplicity	7.0 (7.0)
Completeness (%)^a^ spherical/ellipsoidal	69.2 (14.2)/91.2 (55.2)
Mean I/σ(I)	11.4 (1.5)
*R*‐meas	0.110 (1.354)
*R*‐merge	0.102 (1.256)
*R*‐pim	0.041 (0.503)
CC_1/2_	0.998 (0.569)
Wilson B (Å^2^)	53.72
Refinement	
Resolution (Å)	35.37–2.26 (2.31–2.26)
*R*‐work	0.2178 (0.4774)
R‐free	0.2404 (0.3277)
Number of nonhydrogen atoms, *B*‐value (Å^2^)	
Protein	6053, 63.41
Heterogen	54, 72.85
Solvent	48, 45.45
Rmsd bonds (Å), angles (°)	0.003, 0.518
Ramachandran favored, allowed, outliers (%)	96.18, 3.82, 0.00
Rotamer outliers (%)	1.64
MolProbity clashscore	2.28

^a^ Anisotropic truncation has been used. The first line refers to the spherical and the second line to the ellipsoidal completeness.

### Human subcutaneous adipose tissue collection, isolation of the stroma–vascular fraction, and differentiation of adipocytes

Subcutaneous adipose tissue (SAT) samples were collected during elective aesthetic and body contouring surgery after weight loss surgery at the Division of Plastic, Aesthetic and Special Hand Surgery of University Hospital Leipzig between September and December of 2024. The surgical sites selected for collection were the lower abdomen during abdominoplasty. All surgeries were performed under the influence of general anesthesia. The study excluded patients who received local anesthesia during the procedure. Patients with a history of liposuction or cryolipolysis in the target area were excluded from the study. The subcutaneous tissue was prepared for resection using electrocautery. Thermally damaged tissue and skin were removed, and fat samples were washed with a solution containing Krebs–Henseleit buffer, 2.5 mm CaCl_2_, 25 mm NaHCO_3_, 25 mm HEPES, and 1% BSA. Connective tissue was then removed from the samples, and the fat was processed with scissors to obtain a homogenous mixture. This mixture was then digested with 500 units of collagenase (Type II, Gibco, brand of Thermo Fisher Scientific) per gram of tissue in adipocyte isolation buffer (100 mm HEPES, 123 mm NaCl, 5 mm KCl, 1.3 mm CaCl_2_, 5 mm glucose, 1% CellShield, and 4% BSA) for 45 min at 37 °C. Subsequently, the digested fat was filtered through a 300‐μm syringe strainer (pluriSelect). The mature adipocytes then floated on top, while the infranatant contained the dissociation buffer and the stromal vascular fraction (SVF).

SVF cells were then transferred into a new 50‐mL tube and washed twice with buffer. The resulting pellet was dissolved in red blood cell lysis buffer (154 mm NH_4_Cl, 10 mm KHCO_3_, and 0.1 mm EDTA) and incubated for 7 min at room temperature. Ten milliliters of DMEM/F12 was added, followed by a further 5‐min incubation. The suspension was filtered through a 30‐μm MACS SmartStrainer (Miltenyi Biotec, Bergisch Gladbach, Germany), and preadipocytes were pelleted by centrifugation at 500 ***g*** for 10 min. Following this, the preadipocytes were resuspended in growth medium (DMEM/F12 + 10% FCS, and 1% CellShield). The cells were seeded in 12‐well plates at a density of 300 000 cells per well. Preadipocytes were cultivated in growth medium until reaching a state of confluence (D0). Subsequently, the induction of differentiation was initiated by changing to induction medium (DMEM/F12 + 1% CellShield, 0.5 mm IBMX, 100 nm insulin, 1 nm T3, 100 nm dexamethasone, 1 μm rosiglitazone, 33 μm biotin, 17 μm pantothenate, 10 mg·mL^−1^ transferrin) from D0 to D3. Cells in the D3–D5 stage were subjected to further differentiation with differentiation medium I (induction medium without IBMX), followed by differentiation medium II (DMEM/F12 + 1% CellShield, 100 nm insulin) until D12. Medium changes were performed every 48 h. On D12, cells were subjected to a 30‐min starvation period, followed by incubation with 100 nm TAMRA‐vaspin for 30 min. Following this, the cells were washed with phosphate‐buffered saline (PBS) twice and then snap‐frozen.

### Subcellular fractionation of human adipocytes

Subcellular fractionation was performed by scraping the cells from the wells with 1 mL of PBS + PIC, followed by centrifugation at 4 °C with 300 ***g*** for 5 min. The resultant pellet was washed twice with 200 μL of PBS + PIC. The cells were lysed using 100 μL of NIB + PIC + PI + NP40, and the mixture was incubated on ice for 5 min. Following centrifugation (5 min, 4 °C, 1000 ***g***), the resulting pellet contained the cytosolic fraction. The pellet was washed again with NIB + PIC and centrifuged (5 min, 4 °C, 1000 ***g***) to obtain the remaining cytosolic fraction. The remaining nuclei pellet was resuspended in 30 μL NIB + PIC and briefly sonicated (3 × 10 s) to break the nuclei and obtain the nuclei fraction. A volume of 15 μL of the cytosolic and nuclei fraction sample was used for western blot analysis as previously described [46]. The labeled target protein, vaspin, was identified using rabbit anti‐TRITC antibody (#A6397; Thermo Fisher) and anti‐GAPDH (#G9545; Sigma, St. Louis, MO, USA) or anti‐Histone H3 (#10799; Abcam, Cambridge, UK) as markers for the cytosolic and nuclear fractions, respectively. HRP‐conjugated rabbit/mouse secondary antibody (#7074S, #7076S; Cell Signaling Technologies, Danvers, MA, USA) was used for chemiluminescence detection.

### Fluorescence microscopy

Human coronary artery endothelial cells (HCAEC, from Promocell, Heidelberg, Germany), differentiated mouse 3T3‐L1 (RRID: CVCL_0123, from the ATCC, Manassas, VA, USA) and human SAT SVF‐derived adipocytes were serum‐starved for 30 min prior to the incubation with 100 nm TAMRA‐vaspin and subsequently washed with PBS. Nuclei were stained with DAPI (2 μg·mL^−1^), and fluorescence microscopy was performed using a compact fluorescence microscope BZ‐X800 (Keyence, Osaka, Japan), as previously described [46]. Cell lines were authenticated by STR profiling (Cell Line Authentication Service of Eurofins Genomics, Ebersberg, Germany), and all experiments were performed with mycoplasma‐free cells, regularly tested using the Mycoalert Kit (Lonza, Basel, Switzerland).

### Study approval

Written informed consent was obtained from all patients. The study was approved by the Ethics Committee of the University of Leipzig (approval numbers: 159‐12‐21052012 and 017‐12ek) and performed in accordance with the Declaration of Helsinki, the Bioethics Convention (Oviedo), and EU Directive on Clinical Trials (Directive 2001/20/EC).

### Statistical analysis

Data are presented as mean ± SEM. No statistical comparisons were made in this study.

## Conflict of interest

The authors declare no conflict of interest.

## Author contributions

KM and JTH conceived and designed the study and wrote the manuscript. KM expressed and purified proteins, performed experiments, and analyzed data. AU and NS crystallized proteins and carried out crystal structure determinations. HBe, SB, and MM performed DRaCALA experiments. RN and SL performed abdominoplasties and collected human adipose tissue samples. HBr isolated and cultured human adipocytes. JTH supervised the project. All authors edited and commented on the manuscript and gave final approval for publication.

## Data Availability

All data generated or analyzed during this study are included in this published article.
